# Biopharmaceutical Industry Capability Building in India: Report from a Symposium

**DOI:** 10.1007/s12247-021-09596-9

**Published:** 2021-11-26

**Authors:** Annu Uppal, Ranjan Chakrabarti, Narendra Chirmule, Anurag Rathore, Fouad Atouf

**Affiliations:** 1 US Pharmacopeia India Pvt Ltd, Hyderabad, Telangana India; 2 SymphonyTech Biologics Private Limited, Pune, Maharashtra India; 3 Centre of Excellence for Biopharmaceutical Technology, Indian Institute of Technology, Delhi, New Delhi 110016 India; 4 US Pharmacopeial Convention, Rockville, MD USA

**Keywords:** Quality, Capability, Manufacturing, Biopharmaceutical, Regulatory

## Abstract

The biopharmaceutical industry is evolving with a shift in focus from recombinant proteins and antibodies towards more complex cell and gene therapies. To be competitive globally, biomanufacturers need to focus on aligning with global standards with regard to drug quality, reducing manufacturing failures and delivering drugs to market quickly. Building these capabilities requires a multifaceted approach that includes improvements in operations, quality compliance, and control strategies. To address these needs, the US Pharmacopeia (USP), the Department of Biotechnology (DBT) India, and the Confederation of Indian Industry (CII) held a symposium to discuss the requirements and gaps in the biotechnology and pharmaceutical sectors in India and other developing countries. A panel of experts from academia, manufacturing, and governmental agencies identified several drivers needed for capability building, including a skilled workforce, public–private partnerships, advanced manufacturing technologies, novel biologics, and favorable policies. This article summarizes the recommendations put forward by this panel.

## Introduction

Pharmaceutical companies in middle-income countries such as India and China lead the global generics market, and their contributions have enabled accessibility to affordable quality medicines, especially to the emerging and developed markets. These companies are the largest providers of generic small molecule drugs globally, occupying 40% of the US market and 20% of the global supply share by volume. The COVID-19 pandemic exemplifies this, wherein Indian companies are supplying 62% of the global demand for vaccines [1, 2].

In contrast to the successes in generic drug production, similar achievements for novel and biosimilar biotherapeutic products have yet to be realized due to a couple of major reasons: (i) these biotherapeutics, particularly monoclonal antibodies, are challenging to manufacture, are heterogeneous products, and require a high level of process control for a manufacturer to ensure consistent product quality and (ii) unlike generic drugs, whose price tags typically reflect a 90% reduction from their branded counterparts, a biosimilar is generally sold at a modest 20–30% reduction in cost relative to the innovator drug [3, 4]. One of the reasons for this limited pricing differential is that the manufacturing process for biosimilars and innovator products is more or less the same across the world and quite costly; (iii) the cost-of-goods for a biosimilar ranges from 30 to 50% of the product cost, compared to 5–10% for an innovator drug; (iv) Indian companies tend to stick with the same or similar manufacturing platforms as global innovator companies; and (v) there is reticence for change among Indian drug manufacturers for fear that radical modifications to their manufacturing technologies could increase regulatory risk, result in delays in regulatory approvals, and could even result in regulatory rejection. All these factors contribute to the lack of risk-taking in innovative manufacturing technologies of biotherapeutic products [5, 6].

The generics industry for therapeutics and vaccines has demonstrated significant achievements in the production of cost-effective medicines. For similar success at the upper end of the value chain, there is a need to focus on novel manufacturing and analytical technologies to develop innovative biologics such as monoclonal antibodies, antibody–drug conjugates, and advanced therapies (cell and gene therapies) [6, 7]. For this to be achieved, significant changes in policies and incentives are needed as well as promotion of academic-industry-government collaborations. In addition, there is an urgent need for global meetings in which scientific information with global thought leaders can be exchanged and shared.

This article summarizes discussions held at a symposium co-sponsored by US Pharmacopeia (USP), Department of Biotechnology (DBT), and Confederation of Indian Industry (CII), focused on understanding the existing capability landscape in India and identifying gaps. An expert panel and symposium attendees provided recommendations to strengthen the capabilities of manufacturers with attention on the development of specific human skills needed to spur innovation and establish quality systems in biotherapeutics development programs.^1^

## Proposed Growth Drivers to Strengthen the Capabilities of the Biopharmaceutical Industry

### Skilled Human Resources

**Fig. 1:**
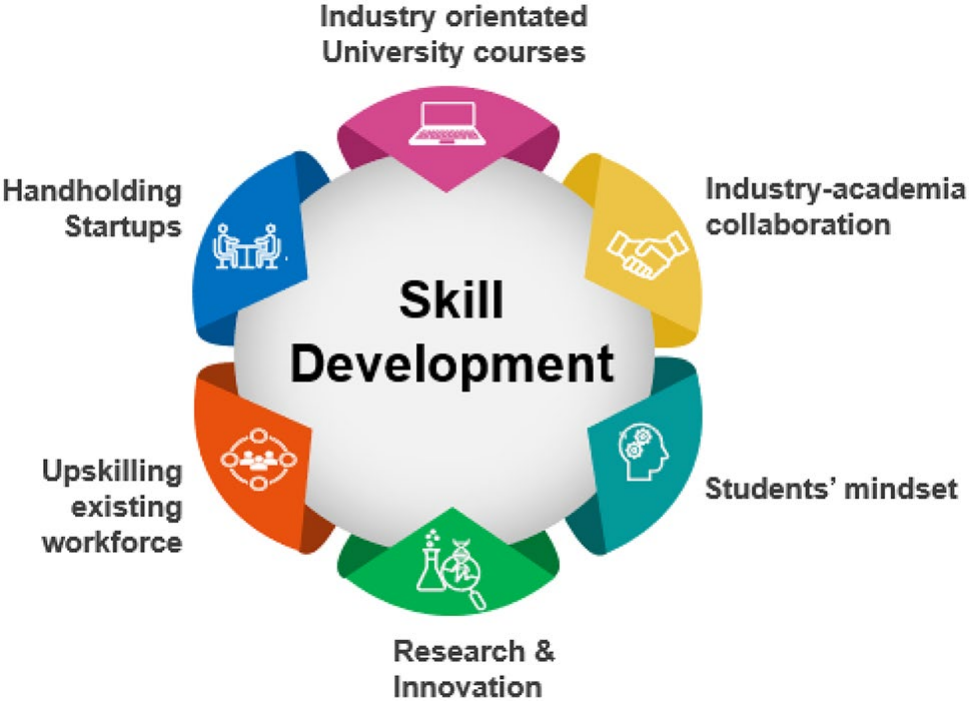
Recommendations for skill development in India

The Indian biopharmaceutical industry is emerging with high-quality, affordable biosimilars entering the developed market. Biocon-Mylan, Intas, USV, and Lupin have biosimilars approved by the US Food and Drug Administration (FDA), European Medicines Agency (EMA), Japan’s Pharmaceuticals and Medical Devices Agency, and other International Council for Harmonisation of Technical Requirements for Pharmaceuticals for Human Use regulatory agencies [8, 9]. These Indian biosimilar manufacturing companies also focus on emerging therapeutics sectors, such as cell and gene therapies and personalized medicines [10, 11]. A focus on innovation-driven research and development (R&D) to promote the growth of the biotherapeutics industry requires the availability of highly and diversely skilled human talent (Fig. 1). Specifically, training needs to be directed towards problem-solving, knowledge of current, cutting-edge technologies that meet global standards, and know-how of quality processes and products. As per a study conducted by the National Skill Development Corporation (NDSC), a not-for-profit nodal agency for skills development and entrepreneurship (Skill India Initiative) under the current Ministry of Finance, a significant skills deficit in human resources in the Indian pharmaceutical industry has been reported [12]. According to the report, India has competitive advantages that favor Foreign Direct Investment (FDI) policies, growth in manufacturing, and a trained labor workforce. However, the elements for a skilled pharmaceutical workforce are lacking.

In the past decade, there had been a significant emphasis on biotechnology courses at all academic institutions. However, the curricula of most institutions at the undergraduate and graduate levels have not been aligned with current industry practices [13]. While subjects relevant to the biopharmaceutical industry — molecular biology, bioinformatics, cancer biology, immunology, biophysical and biochemical techniques — are a part of the curriculum, they would be more valuable if taught real-life case studies by industry experts. Among the challenges of this is the lack of time among industry personnel to provide such dedicated teaching, and limited demonstration by universities to create flexible, mutually beneficial environments to enable such collaboration. Understanding the process of drug development requires an inter-disciplinary approach that spans the basic sciences of biology-chemistry-physics, chemical engineering, pharmacology, veterinary sciences, clinical studies, and economics and law. To attract top talent from academia and overseas, the industry needs to focus on innovation, which will also help them grow their value chain. Consequently, greater emphasis on collaboration that would promote diversity in thought and merge interdisciplinary fields will prepare students and faculty to contribute towards progress in innovation in the biopharmaceutical industry [13].

Equally important is the continued professional development and upskilling of the current workforce. It was recommended that a focus on skills needed for biologics manufacturing would be beneficial. Areas include analytical, good manufacturing process and quality management, documentation, regulatory, digitization, and big data analysis [14]. With the adoption of newer manufacturing technologies that include automation and digitization with real-time information gathering, enhanced training of the biopharma workforce is required to analyze data effectively and draw scientific conclusions. There are multiple programs run by numerous institutions/authorities and government to bridge these skills gaps, but these programs run in silos [15]. Convergence of these training programs or a centralized system to certify these training before they are delivered is an urgent need to develop a skilled workforce. It is also important that the Government of India work with global organizations like the USP that has expertise in Quality System requirements and advanced technology for the development of quality products for developed markets. This type of collaboration can help to bring such a knowledge base to India to train scientists.

It is also important that regional regulators are properly trained to ensure the quality of the products they are approving [16]. Regulators of low-and-middle-income countries (LMIC) often face challenges in making sure that medicines are manufactured, imported, stored, distributed, and utilized according to regulated market standards. Collaboration with organizations having experience with advanced world regulatory processes can also help in this aspect. An example is the PQM + program (2019–2024) which is funded by the US Agency for International Development and implemented by the USP and its consortium of partners. This program is designed to build capabilities in LMIC by providing technical assistance to manufacturers and support to Medicines Regulatory Authorities to advance global learning and policy making, and strengthen the quality assurance systems by applying international quality standards [17, 18]. Importantly, PQM+ work with local and regional organizations will help to build on-the-ground specialized expertise where it is needed most. This includes accreditation of national drug quality control laboratories per International Organization for Standardization (ISO) 17025 and World Health Organization prequalification standards.

### Innovative Models of Academic — Industry Partnerships

Examples of fruitful partnerships between industry and academia are far and few in between [19]. The primary reason for the limited collaboration has been industry and academia’s fundamentally different drivers and motivations. To a large extent, the industry is driven by near-term outcomes such as investments in projects that can be rapidly implemented for profitable results. A survey of industry-funded projects by major Indian academic institutions shows that most industry-funded projects are of 1–2-year durations with many < 1-year duration and very few of > 3-year periods.

In contrast, academics wish to pursue novel ideas and approaches through the proof-of-concept stage. They feel burdened to take an idea further into validation and eventually implementation in the industry. At best, most academic research efforts conclude with a good publication, rarely with a technology that is adopted by industry or a commercial product [20]. There are two major hurdles in accomplishing the above as they are in contrast to the current academic environment and infrastructure. First, a significant amount of time is required to take a proof-of-concept stage product to commercialization onto the market. For example, a biotherapeutic product can take more than 10 years to reach the market. Most academics are unable to provide the sustained effort required for such a length of time. Second, bringing a commercial product to market often requires a multitude of expertise and steps that depend on various entities. For example, bringing a biotherapeutic product to the market requires rigorous non-clinical and clinical trials followed by regulatory approval. This necessitates collaboration between the academic who creates a novel product and industry whose participation is necessary to take the product through manufacturing, clinical testing, regulatory approval, and commercialization [20].

As a result, the Indian industry has traditionally been in-licensing technology from global innovators [21]. This practice has hurt the local industry as this dependency has resulted in the use of old technologies, with only a few biopharma companies having credible and modern in-house research efforts. More recently, the Indian government, industry, and academia have taken note of the lost opportunity. Most of the major funding agencies, such as the Department of Science and Technology (DST) and the DBT have been sponsoring programs that require industry participation to receive funding [22]. An example of such a scheme is the Uchchatar Avishkar Yojana (UAY), a program launched by the Prime Minister’s office. The UAY program requires that proof-of-concept has been already established for a technology and that at least 25% of the funding come from an industry partner [23]. This has served two critical purposes: (i) the requirement for industry funding acts as a screening step as projects with low chances for commercialization are unlikely to find an industry partner willing to fund them and (ii) after funding a project, the industry partner would likely be interested in taking the product to the market, thereby spurring a commercialization partner to be in place. As a result, the UAY example has the potential to be a successful program for generating fruitful academia-industry collaborations.

As more such collaborations succeed, much-needed cultural changes are likely to follow. Academics should increasingly become more interested in taking their research further to co-create technologies and products with commercial potential. Industry, too, is expected to see value in these collaborative relationships and view academia as a source for not only novel ideas, technologies, and products, but also as a means to strengthen their in-house research efforts, a must if “Make in India” is to be a success. A major positive change that has been demonstrated in the last decade has been the rising interest in start-ups on academic campuses. Primarily driven by students, with healthy involvement of academic faculty, institutions across India are buzzing with start-up activities. These start-ups are also becoming paths for academic-industry collaborations. Increasingly, the industry has been willing to invest in these start-ups and in the process, accelerate the commercialization of start-up technologies. India is already the 3rd largest start-up ecosystem in the world with over 4237 biotech start-ups, nearly a 25% jump since 2019 [24]. This data suggests that the emergence of successful models of academia-industry interactions is on the horizon.

### Improving Manufacturing Technology for Biopharmaceutical Production

Generics and vaccine manufacturing companies have already established themselves as successful manufacturers of safe, effective, and economical pharmaceutical and vaccine products. Adopting advanced manufacturing technologies, significant investment, and enhanced infrastructure capacity is required to replicate this to the novel biopharmaceutical sub-sector. Continuous processing, for one, has emerged as the technology of the future [25, 26]. It involves running unit operations of a process at steady state such that production can continue for a considerable amount of time (typically in the months). This is different from batch production, where the material moves along stage by stage, with discrete batches of product produced, and with each batch typically requiring weeks to be produced. Many industries have successfully migrated from batch to continuous processing including the petroleum, steel, automobile, and fast-moving consumer goods (FMCGs) industries. However, the biopharmaceutical industry has yet to do so. Over the last 10 years, considerable work has been done in this field by both academia and industry. It has been demonstrated that switching from batch to continuous processing can increase productivity by about 10× and reduce the cost of goods by 50–70% [27]. These benefits are realizable based on the need for a significantly smaller facility (and thereby reduced capital cost), improved facility utilization, streamlined process flow, improved process control, and more consistent product quality. Regulators are also quite supportive of this transition. Both the US FDA and EMA have encouraged manufacturers to make this transition. Concerning technology, the use of perfusion reactors for high-density cell cultures has been extensively demonstrated over the last two decades. In the previous 5 years, feasible solutions that enable key unit operations such as process chromatography, refolding, precipitation, cell lysis, filtration, and aqueous two-phase extraction in a continuous fashion have also been demonstrated. Indian companies need to aggressively pursue the adoption of continuous processing, as this will aid in their emergence as competitive, global manufacturers of novel biopharmaceutical products.

Two other major trends accompanying the rise of continuous manufacturing are automation and digitization [28]. When a manufacturer transitions from batch to continuous manufacturing, monitoring and process control dramatically change. Despite the drawbacks, batch manufacturing has the advantages of ease with in-process data review and analysis and decision-making capability regarding the next steps before initiating the next process step. Continuous processing takes this flexibility away. If there is a breakdown in the process, the manufacturer will have no choice but to stop the process, clean the entire production line, and restart. Such break-downs, if too frequent, threaten to take away all productivity gains mentioned above. Even process variability, if too much, can result in the process spiraling out of control. Thus, the successful operation of continuous processes requires more robust process controls than batch processes. The creation of such control schemes requires heavy use of automation and digitization [29]. Appropriate analytical tools must be identified to gather information about the desired product attributes, process attributes, or process parameters. Real-time monitoring is desirable, and this has resulted in the emergence of spectroscopy-based methods for process monitoring. This data then needs to pass to the controller, often based on a mechanistic, empirical, or hybrid unit operation model. The controller’s output subsequently needs to go back to the process or analytical equipment manager as the control scheme requires. The overall control scheme needs to work in an automated manner, immune from manual interventions, and mistakes. However, once developed and implemented, continuous processing offers a more economically viable and productive path than batch processing.

Thus far, generic and biosimilars manufacturers have been followers of US- and EU-based innovators. These manufacturers need to adopt a culture of innovation, which will result in emerging and underdeveloped economies being major beneficiaries. Although the initial investment is high to enable this, it has been shown that the implementation of novel technologies, in the long run, will also save money and time [28]. This should encourage cost-conscious Indian companies to adopt such technologies.

### Innovation and Development of Novel Biologics

Generics and vaccine manufacturers have been very successful in developing cost-effective medicines and thereby making them affordable globally. To become a hub for cutting-edge innovation and research, Indian manufacturers need to focus efforts on developing innovative products and technologies, especially for diseases of significant unmet need [30]. Currently, India’s innovative pharmaceuticals risk/reward index is far behind at 54.6/100. The country’s standing is behind not only to that of developed nations (global rank of 45 out of 109), but also to several Asian counterparts in this area (regional rank of 11 out of 20) [31]. Adoption of new technologies in biotechnology (genomics to gene editing) and digitization (block-chain, 5G, quantum computing, machine learning) can drive innovation but will require an exponential increase in R&D investments and collaborations [32]. This has been recognized by the government, which has recently rolled out several initiatives such as “Make in India” and “Start-up India,” that look towards building an innovation ecosystem to encourage domestic R&D capabilities and attracting global investments. The Department of Science and Technology-Science and Engineering Board (DST-SERB) has announced special research projects to ramp up national R&D efforts against the COVID-19 pandemic [33]. DST-SERB has put together a special expert committee, which has selected five projects whose technologies will help find effective measures to combat infectious disease. They are also providing funding to the industry to promote innovation and research in the development of indigenous COVID-19 vaccine candidates. However, to move up the value chain with innovative products, the government needs to increase its investments in the necessary R&D infrastructure, education of a skilled workforce, promotion of academic-industry collaborations, and the development of a suitable regulatory framework.

It has been established that a robust academic system is required to promote innovation and research output. In the USA, Europe, and certain Asian countries, where innovation is prevalent, most innovative ideas stem from academia, whose proof-of-concept studies are taken up by start-ups for further development. In turn, start-up outputs are subsequently taken up by industry for further development and, ultimately, commercialization [24]. Thus, cross-functional collaborations with inter-disciplinary approaches are required to take innovative drugs from bench to bedside, requiring academia, start-ups, industry, and government collaborations. The drug development process spans the discovery of disease biology and mechanism of action studies to process and product development, pharmacology, toxicology, and clinical trials. The process requires specialization and experience in various areas of science, and a breadth of knowledge spanning the entire development process along with significant funding. Most academic institutions are not familiar with the complexity of drug development, as it is not part of their curriculum.

Engagement and collaboration with the Global Indian Diaspora to take advantage of their potential and experience in translational science can contribute to the development of novel biotherapeutics. To be successful in this area, we need to take few concrete steps:

- Build an innovation ecosystem in which both academics and industry co-create an environment that promotes, protects, and commercializes research to attract and retain top talent. Today, most of our top talent goes abroad because of the lack of R&D infrastructure and funding to facilitate innovative research.
- Our government should provide sufficient funding throughout the innovation life cycle.
- Academic institutions should provide an amenable environment, the freedom to bring talent back to India, and assist them in pursuing innovation and effective industry collaborations.
- The government needs to provide enough incentives to industry, like in China, Korea, and Singapore, to encourage them to invest in drug discovery. The Indian pharmaceutical industry is generally risk-averse and unfortunately innovative research has a high risk of failure.
- Greater involvement by industry in nurturing start-ups, picking up promising, innovative products for the next development stage, with the help of government funding. Today, the Indian government has started several programs to fund start-ups and has contributed to their significant growth, with over 4237 biotech start-ups presently, nearly a 25% increase since just 2019 [24]. But, unfortunately, most of the ideas die after proof-of-concept due to a lack of understanding of the R&D, regulatory, and commercialization process [20].

Thus, to make India an innovative hub, all stakeholders must work together to build, sustain, and grow a scientific, economic, and policy ecosystem that promotes and rewards biological innovation.

### Role of Policy Frameworks

Regulatory agencies play a major role in the governance of the drug development process. A strong, transparent regulatory framework is essential for the growth of biopharmaceutical industries in a global, competitive environment. The Indian government has been increasing investment, attracting multinational biopharmaceutical companies and improving the Indian business environment. The recently launched Production Linked Incentive (PLI) program is an excellent example of such steps taken by the government to encourage R&D base setup and support for domestic companies to establish new facilities or expand existing manufacturing units [34]. However, there remain opportunities to strengthen the regulatory system to avoid delays in regulatory processes. To promote the innovation ecosystem and faster approval of innovative drugs, the adoption of new policies or suitable amendments to the current regulatory path are required to improve processes and fast-track approvals, especially in the case of novel therapies like cell and gene therapies. The government needs to ensure that technically trained people are managing these advanced areas in regulatory bodies. In addition, there is a need to expedite and streamline regulatory paths for import–export permits for biological samples, especially for advanced cell-based therapies, to reduce the approval time to the benefit of patients. Further, policy support for technology transfer and investments in R&D is needed by academia and start-ups to enable scale-up of their early research to production under compliant environments in collaboration with industry [35].

In India, there are multiple bodies regulating recombinant products [36]. The Central Drugs Standard Control Organization (CDSCO), headed by the Drug Controller General of India (DGCI), regulates pharmaceutical and medical devices under the auspices of the Ministry of Health and Family Welfare. The DCGI is advised by the Drug Technical Advisory Board (DTAB) and the Drug Consultative Committee (DCC). The regulation of safety under preclinical toxicology is governed by the Review Committee of Genetic Manipulation (RCGM), which falls under the Department of Biotechnology. This complete governance process includes a three-tier review committee comprising the Institutional Biosafety Committees (IBSC) at the institute/company, the RCGM in the Department of Biotechnology, and the Genetic Engineering Approval Committee (GEAC) in the Ministry of Environment and Forests (MoE&F) for monitoring, evaluation, and approval granting for research and development activities on recombinant DNA products. This governance requires efficient collaboration across the various regulatory bodies to provide a framework for establishing checks and balances in the approval of medicines. It will benefit the industry if the above process is streamlined and a single body is responsible and accountable for the end-to-end governance.

To strengthen the regulatory system and enhance regulator capabilities, it is thus critical to ensure better coordination among agencies, increased resources and training, and policy reforms. These modifications will help support the innovation and research ecosystem for meaningful drug research that can address the healthcare needs of India and the global population.

## Summary and Recommendations

**Fig. 2:**
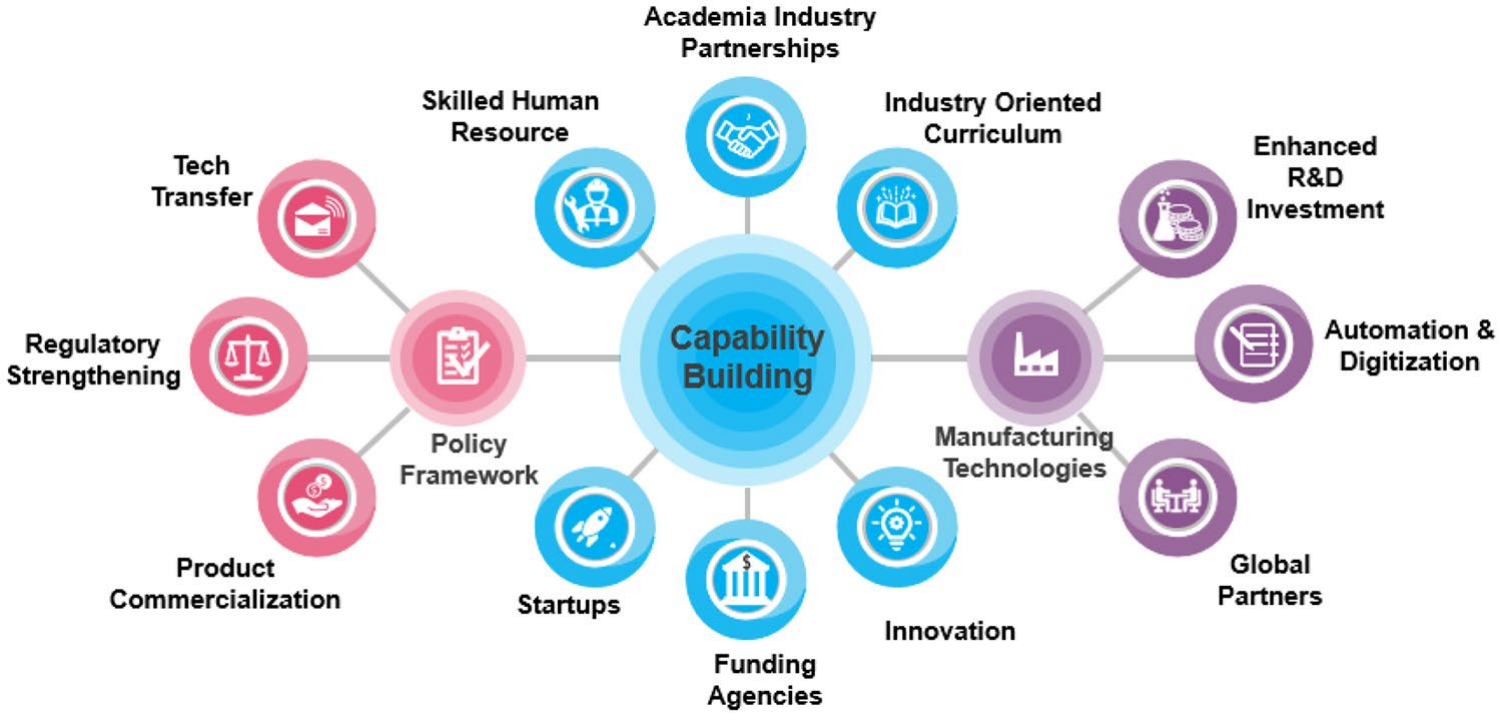
Capability building landscape for biopharma industry

In summary, the path for the success of the Indian biopharmaceutical industry depends on collaboration and innovation supported by new policies (Fig. 2). If numerous competitive advantages in R&D facilities, knowledge, skills, and cost effectiveness were supported, the biotechnology industry in India could have immense potential to emerge as a global key player.

The following recommendations should be implemented to enable the success of the Indian biopharmaceutical industry:

Workforce skills development:

1. Update biotechnology curriculum in alignment with industry requirements with a focus on drug discovery and translational research.
2. Skills development in emerging technologies, management, and entrepreneurship and support for interdisciplinary skill sets aligned with global standards.
3. More interactions between academics (students and faculty) and industry, and vice versa. Provision for industry training or internship for graduate/post-graduate students to enhance R&D skills. Industry must send their staff to academia for such teaching/training.
4. Need for programs co-sponsored by government and industry to bring back Indian PhD postdocs to India to work on challenging industry projects to generate more innovation and intellectual property for the country.
  Academia industry collaborations:
5. Crosstalk between academia-industry is needed to promote innovative research on industry-specific topics.
6. Shared resources or high-end R&D centers to promote accessibility of latest technologies to start-ups and small-scale industries.
  Manufacturing technologies:
7. Increase in R&D investment/incentives by the government to academics and industry to adopt advanced technologies like continuous manufacturing, digitization, and automation.
8. Enhanced engagement and collaboration with Indian diaspora scientists from top universities and companies to leverage their experience.
  Innovation:
9. A requirement for partnership in intellectual property (industry and academia).
10. Enable technology transfer to facilitate the flow of academic research to companies for further development and commercialization.
11. More handholding and strengthening of the start-up system would generate awareness about funding, regulatory guidelines, and product commercialization. Large industries to come forward to take risks, invest in early stages of innovation that would take the technologies forward.
  Policy framework:
12. Review regulatory frameworks to ensure a more rapid introduction of innovative products into the market.
13. Continuous and up-to-date education for regulators in alignment with emerging technology requirements.

## Data Availability

The datasets/research used during the current study are available from the corresponding author.

## Footnotes

1 Koduru V Surendra Nath USP; Meenakshi Munshi DBT; Ranjit Madan LSSSCD; Anurag Rathore IIT D; Sanjay Singh Genova; Reba Chhabra, NIB;; Narender Chirmule Symphonytech biologics; Gopala Dasika Biocon; Sridevi Khambhampaty Intas; Dhaval Trivedi Syngene, Mirage Singh, DBT, Ranjan Chakrabarti USP, Annu Uppal, USP.
